# Perinatal Asphyxia Reduces Dentate Granule Cells and Exacerbates Methamphetamine-Induced Hyperlocomotion in Adulthood

**DOI:** 10.1371/journal.pone.0003648

**Published:** 2008-11-05

**Authors:** Tomoyasu Wakuda, Hideo Matsuzaki, Katsuaki Suzuki, Yasuhide Iwata, Chie Shinmura, Shiro Suda, Keiko Iwata, Shigeyuki Yamamoto, Genichi Sugihara, Kenji J. Tsuchiya, Takatoshi Ueki, Kazuhiko Nakamura, Daiichiro Nakahara, Nori Takei, Norio Mori

**Affiliations:** 1 Department of Psychiatry and Neurology, Hamamatsu University School of Medicine, Shizuoka, Japan; 2 Department of Anatomy and Neuroscience, Hamamatsu University School of Medicine, Shizuoka, Japan; 3 Department of Psychology, Hamamatsu University School of Medicine, Shizuoka, Japan; 4 Research Center for Child Mental Development, Hamamatsu University School of Medicine, Shizuoka, Japan; 5 Research Center for Child Mental Development, Graduate School of Medicine, Osaka University, Osaka, Japan; 6 The Institute of Psychiatry, London, United Kingdom; Chiba University Center for Forensic Mental Health, Japan

## Abstract

**Background:**

Obstetric complications have been regarded as a risk factor for schizophrenia later in life. One of the mechanisms underlying the association is postulated to be a hypoxic process in the brain in the offspring around the time of birth. Hippocampus is one of the brain regions implicated in the late-onset dopaminergic dysfunction associated with hypoxic obstetric complications.

**Methodology/Principal Findings:**

We used an animal model of perinatal asphyxia, in which rat pups were exposed to 15 min of intrauterine anoxia during Cesarean section birth. At 6 and 12 weeks after birth, the behavior of the pups was assessed using a methamphetamine-induced locomotion test. In addition, the histopathology of the hippocampus was examined by means of stereology. At 6 weeks, there was no change in the methamphetamine-induced locomotion. However, at 12 weeks of age, we found an elevation in methamphetamine-induced locomotor activity, which was associated with an increase of dopamine release in the nucleus accumbens. At the same age, we also found a reduction of the dentate granule cells of the hippocampus.

**Conclusions/Significance:**

These results suggest that the dopaminergic dysregulation after perinatal asphyxia is associated with a reduction in hippocampal dentate granule cells, and this may partly contribute to the pathogenesis of schizophrenia.

## Introduction

Perinatal asphyxia, which may affect 2–4 neonates per 1,000 births, is a major public health concern, especially when it occurs in preterm neonates. During this type of labor complication, the brain will be protected from ischemic damage to as great of an extent as possible. Shankaran and colleagues [1] demonstrated that although a high proportion of neonates suffered from severe anoxia affecting multiple organs they showed no overt signs of brain involvement. Nonetheless, epidemiological evidence suggests that obstetric complications, particularly those related to hypoxia during labor and delivery, may be a risk factor for the development of schizophrenia later in life [2]–[5].

The issue of perinatal asphyxia has been studied in a rodent model of global hypoxia during Caesarean section (C-section) birth [6]. In this model, the intact uterus containing rodent pups is isolated from anesthetized dam by a C-section on the expected day of birth and immersed in a water bath kept at 37°C for 14–17 min for induction of intra-uterine global hypoxia. Neonatal rats born by C-section with global hypoxia have increased numbers of tyrosine hydroxylase-immunoreactive cell bodies in the substantia nigra and ventral tegmental area [6]. Boksa and co-workers have extensively characterized this model and clearly indicated that C-section birth itself is sufficient to produce long-term changes in dopaminergic parameters in rats, such as amphetamine-induced locomotion, expression of dopamine receptors and transporter in basal ganglia, and tyrosine hydroxylase mRNA expression in the nucleus accumbens [for review, see ref. 7]. In addition, guinea pigs born by C-section with global hypoxia show a deficits in prepulse inhibition (PPI) of acoustic startle response [8], although there have been no report describing PPI deficits in rats of this model to date. In most of these previous studies, the C-section was performed in decapitated dams. Under such conditions, C-section birth alone can cause a reduction in systemic oxygenation due to the decapitation [9]. These findings suggest that exposure to hypoxic events (induced by C-section procedure and/or intrauterine asphyxia) during prenatal period in experimental animals can cause long-term alterations in dopaminergic system, which are characteristics implicated in pathogeneses of schizophrenia.

Hippocampus is one of the brain regions which play important role in regulation of mesolimbic dopaminergic function. Previous studies have shown that lesion placements in the bilateral hippocampi, induced by aspiration [10], [11], or intrahippocampal injection of either ibotenic acid [12] or combined kainic acid/colchicines [13], [14], has been shown to greatly enhance locomotor activities induced by an indirect dopamine agonist amphetamine in rats. Furthermore, schizophrenic patients who have been exposed to adverse pre- or perinatal factors are likely to show decrements in hippocampal volume [15], [16]. Therefore, hypoxic obstetric events may impair the development of temporal lobe structures, especially the hippocampus, although underlying mechanisms are unclear. As to the animal model of perinatal asphyxia, a previous study examined the histology of brain regions including hippocampus at adulthood by measuring neuron density [17] and showed a loss of neurons in the CA1 of hippocampus in adult rats experienced C-section with global hypoxia. However, neuron density is strongly influenced by size, shape, and orientation of the neurons and by alteration in volume of the region.

The aim of the present study is to examine whether hippocampal neurons were reduced in adult rats that had experienced perinatal asphyxia during C-section birth, and, if so, whether the reduction of hippocampal neurons related to a dopaminergic dysfunction. We obtained animals born by C-section with or without added global hypoxia, and examined at two developmental periods, adolescence (6-week-old) and adulthood (12-week-old). The dopaminergic function was assessed by a methamphetamine-induced hyperlocomotion. In a parallel experiment, pyramidal neurons in the CA1 and CA3 regions and granule cells in the dentate gyrus of the hippocampus were quantified by a stereology in order to shed light on the roles played by this structure in late-onset changes in dopaminergic function following perinatal asphyxia.

## Materials and Methods

### Animals and induction of perinatal asphyxia

All experiments were performed in accordance with the Guide for Animal Experimentation at the Hamamatsu University School of Medicine. Intrauterine anoxia was induced in rats delivered by C-section according to the method described originally by Bjelke and colleagues [6]. Pregnant female Sprague-Dawley rats (Japan SLC, Hamamatsu, Japan) within the last day of gestation were anesthetized by diethyl ether and hysterectomized. The uterus, including fetuses, was placed in a water bath at 37°C to induce 15 min of asphyxia, which is associated with 100% survival [18]. After delivery, the umbilical cord was ligated and the pups were left to recover on a heating pad for at least 40 min. Rats that had delivered normally were used as surrogate mothers, and their pups were used as vaginally delivered pups. Each surrogate mother received four pups from another surrogate mother, four C-section-delivered, and four asphyxia-exposed pups. At 3 weeks after birth, male rats were selected for the experiments described below and were housed three per cage in a temperature- and humidity-controlled colony room, which was maintained on a 12-h light/dark cycle (07:00 to 19:00 h light on) and with food and water provided *ad libitum*. The animals were divided into 3 groups based on the circumstances of their delivery: vaginal delivery (V group), C-section (C group), or C-section with 15 min of perinatal asphyxia (A group).

### Behavioral analyses

#### Motor activity

Horizontal locomotor activity was measured in 6- and 12-week-old animals by means of an infrared-beam, passive-sensor system (SCANET-SV20; Melquest Ltd., Toyama, Japan). The system consisted of a rectangular enclosure (480×300 mm^2^). The walls of the enclosure were equipped with 144 pairs of photo sensors located at 5-mm intervals. A rectangular, transparent-plastic cage (internal floor area, 440×260 mm^2^; height, 400 mm) was set on the SCANET system such that the photo sensors were 30 mm above the floor of the cage. A pair of photo sensors was scanned every 0.1 s to detect locomotion. An intersection of four consecutive pairs of photo sensors (20 mm apart) in the enclosure was considered as a single unit of locomotor activity. In order to assess the basal activity in each group, animals were placed individually in the observation cage at 16:00. The spontaneous locomotor activity of the animals was monitored during the dark period between 19:00 and 07:00, and then the animals were returned to their home cage. Three days later, each animal was weighed and placed alone in the observation cage a second time, and the animal's locomotor activity was again monitored. After a 60-min acclimation period, the animals were injected intraperitoneally (IP) either with vehicle (saline) or with methamphetamine (at doses of 0.5- or 2.0-mg/kg; d-methamphetamine, Dainippon Pharmaceuticals, Ltd., Osaka, Japan) at a volume of 1.0 ml/kg, and locomotor activity was measured for an additional 90 min.

#### *In vivo* microdialysis

Under pentobarbital anesthesia (50 mg/kg, IP), 11-week-old animals underwent implantation of a stainless-steel guide cannula equipped with a dummy probe (i.d. 0.24 mm, o.d. 0.46 mm; Plastics One, Roanoke, VA, USA) over the right nucleus accumbens; the coordinates were AP 1.2, ML 1.5, and DV 5.5 with respect to the bregma, according to the atlas of Paxinos and Watson [19]. Two additional screws were placed on the skull and served as anchors, and the entire assembly was attached to the skull with dental acrylic. A removable stainless-steel stylet was placed into the guide cannula. Seven days later, when the animals reached 12 weeks of age, the dummy probe was replaced with a dialysis probe (membrane length, 2 mm; o.d., 0.22 mm; wall thickness, 10 µm; EICOM, Kyoto, Japan) and the animals received a perfusion (1 µL/min) of artificial cerebrospinal fluid via the probe. Each animal was injected IP with vehicle at 60 min after the initiation of perfusion, and, 60 min later, given methamphetamine (2.0 mg/kg, IP). Dialysis samples were collected every 30 min for 240 min. The amount of dopamine in each collection was measured by high-performance liquid chromatography coupled with electrochemical detection, as reported previously [20]. After the experiments were carried out, the correct placement of the probes was evaluated by Nissl staining.

### Stereological analysis

An independent group of animals that did not undergo behavioral tests of methamphetamine-induced locomotion was subjected to morphological analysis at 6 and 12 weeks of age. All animals were deeply anesthetized with pentobarbital (100 mg/kg, IP) and sacrificed by transcardial perfusion with 4% paraformaldehyde. The brains were sliced into 30-µm serial coronal sections using a cryostat, and a series of sections was Nissl-stained to obtain a principal neuron count. The stereological procedure was conducted using a computerized system (StereoInvestigator; Microbrightfield Japan, Inc., Chiba, Japan) and Olympus BX40 microscope. The volume of the dentate granular layer as well as that of the pyramidal cell layers CA1 and CA3 were estimated according to Cavalieri's principle [21]. For the cell count, corresponding areas were sampled in a systematic random fashion according to the optical fractionator method. The principal cells were counted in 3-dimensional counting frames using a sampling grid of 180×180 µm for the dentate gyrus and for the CA1 and CA3 regions. The cell-count frame size was 15×15×10 µm for the dentate gyrus and the CA1 region, and 25×25×10 µm for the CA3 region. The number of cells was counted in the right hemisphere of every sixteenth sections in the case of the dentate gyrus, and of every eighth sections in the case of the CA1 and CA3 regions. Sectioning and cell counting were performed by separate investigators who were blinded with respect to the group to which the animals belonged.

### Statistical analysis

The data are expressed as the means±standard errors of the means (SEM). Data from methamphetamine-induced locomotor activity analyses were compared by two-way (birth groups and doses of the drug) analysis of variance (ANOVA). Changes in dopamine release in the nucleus accumbens before and after the methamphetamine administration were compared by one-way repeated measures ANOVA. Other comparisons of data from the three experimental groups were done by one-way ANOVA. If required, post-hoc comparison by Bonferroni test was performed. All the statistical analyses were performed using statistical software (SPSS version 12.0J). The level of significance was set at p<0.05.

## Results

### Behavioral analyses

#### Motor activity

**Figure 1:**
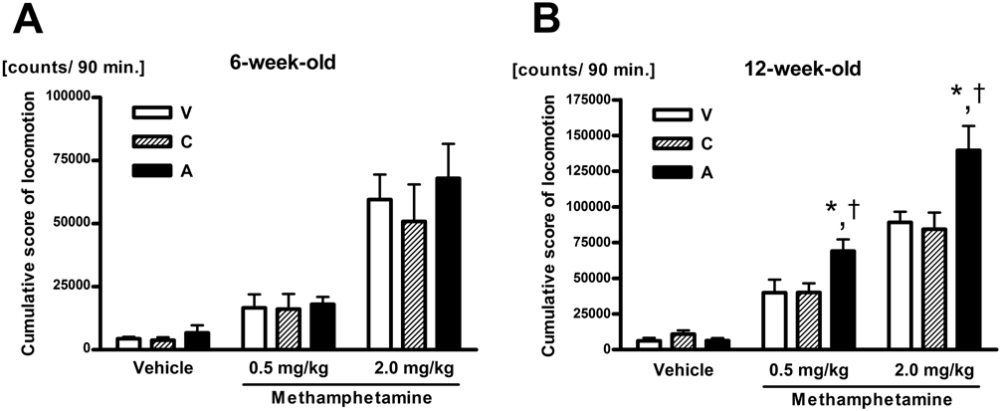
Effects of C-section with global asphyxia on methamphetamine-induced locomotor activity. Vehicle- or methamphetamine-induced locomotor activity in animals from the V (open column), C (hatched column) and A (closed column) groups during a 90-min observation period is summarized for each experimental period: 6 weeks (A, n = 8 for each column) and 12 weeks (B, n = 10 for each column) after birth. Values are expressed as means+SEM. Significant differences: *p<0.01 vs. the V group; †p<0.01 vs. the C group.

During both the 12-h period in darkness and the 60-min acclimation period before the administration of drugs, at both 6 and 12 weeks, animals of each of the 3 birth groups exhibited similar locomotor activity (data not shown). The body weight of the animals at the time of measurement of methamphetamine-induced locomotion is shown in Table 1. The A-group animals tended to weight less than animals from the other groups, but the difference was not statistically significant [F(2 69) = 2.43, p = 0.10 at 6 weeks; F(2 87) = 2.92, p = 0.06 at 12 weeks]. The cumulative scores for 90 min after injection of vehicle or methamphetamine (0.5 or 2.0 mg/kg) are shown in Figure 1. There was no significant difference among the 3 birth groups at six weeks of age, there was no significant effect of birth group [F(2 63) = 0.42, p = 0.66] on the magnitude of locomotor activity, and no interaction between birth group and methamphetamine dose [F(4 63) = 0.10, p = 0.98] was observed (Figure 1A). However, in 12-week-old animals, there was a significant effect of birth group [F(2 81) = 8.82, p<0.001] on locomotor activity, and a significant interaction of birth group and methamphetamine dose [F(4 81) = 3.24, p<0.05] (Figure 1B). When compared with the corresponding value of the V and C groups, the A group exhibited a significantly greater amount of locomotor activity after 0.5 and 2.0 mg/kg of methamphetamine (p<0.01 for both doses).

**Table 1:** Effects of C-section with global asphyxia on body weight.

Age	Groups	Body weight (g)
6 weeks	V (n = 24)	151.9±5.6
	C (n = 24)	151.3±4.9
	A (n = 24)	139.2±2.9
12 weeks	V (n = 30)	367.0±6.2
	C (n = 30)	356.3±4.1
	A (n = 30)	350.2±4.4

There were no significant body-weight differences among the 3 groups. Data are presented as means±SEM.

#### *In vivo* microdialysis

**Figure 2:**
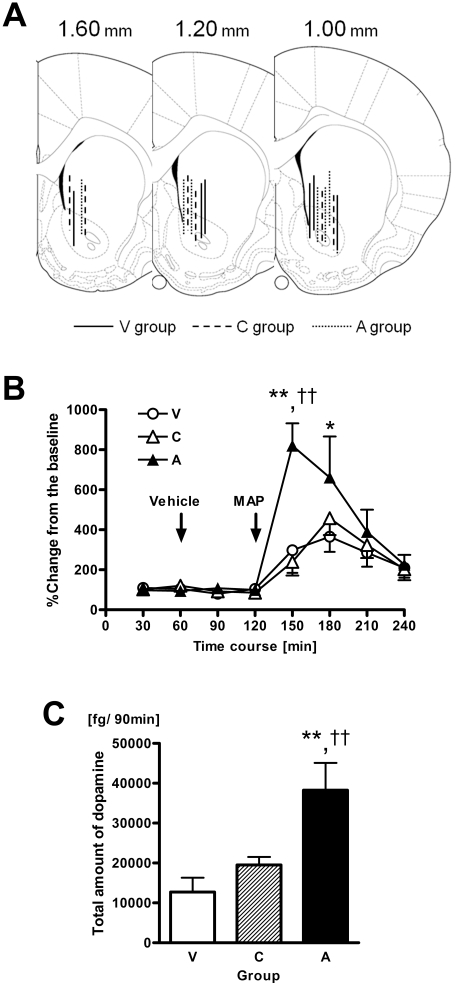
Effects of C-section with global asphyxia on methamphetamine-induced dopamine release. (A) Illustration of the location of dialysis probes in the nucleus accumbens. Lines indicate probe tracks within the nucleus accumbens of animals from groups V (solid lines, n = 6), C (broken lines, n = 6), or A (dotted lines, n = 6). (B) Vehicle- or methamphetamine-evoked dopamine release in the nucleus accumbens in V (○), C (▵) and A (▴) group animals at 12 weeks after birth (n = 6 for each of the V, C, and A groups). The symbols and bars represent means±SEM. Dopamine release is expressed as the %change relative to the baseline. (C) The total amounts of methamphetamine-evoked dopamine release in the nucleus accumbens in the V (open column), C (hatched column), and A (closed column) groups during a 90-min period are summarized. Values are expressed as means+SEM. Significant differences: *p<0.05 and **p<0.01 vs. the V group; ^††^p<0.01 vs. the C group.

We measured the extracellular amount of dopamine in the nucleus accumbens after the administration of 2.0 mg/kg methamphetamine to 12-week-old animals. It was verified that the dialysis probe had been correctly placed within the nucleus accumbens in all animals (Figure 2A). The basal level of extracellular dopamine was similar among the 3 birth groups [F(2 15) = 1.22, p = 0.31] (Figure 2B). Vehicle injection did not alter the dopamine levels [F(2 15) = 0.79, p = 0.46] (Figure 2B). One-way repeated measures ANOVA revealed a statistically significant interaction between Group and Time [F(14 91) = 2.51, p<0.01]. The *post-hoc* Bonferroni tests revealed that the elevation in dopamine release in animals from the A group was significantly larger than that of both the V and C groups (p<0.01 for both groups) during the 30-min period after the injection of methamphetamine, and the elevation in dopamine release was also greater in the A group than in the V group (p<0.05) in the period from 30 to 60 min after the injection of methamphetamine (Figure 2B). The total amount of dopamine within the 90-min methamphetamine post-injection period in the A group was significantly higher than that of the V and C groups (p<0.01 for both groups) (Figure 2C).

### Stereological analysis

The results of the stereological analyses of the hippocampus are summarized in Table 2. Among animals at either 6 or 12 weeks of age, there were no significant birth-group differences in terms of the estimated volume of the CA1 region [F(2 21) = 0.22, p = 0.81 at 6 weeks; F(2 20) = 1.29, p = 0.30 at 12 weeks], the CA3 region [F(2 21) = 0.11, p = 0.97 at 6 weeks; F(2 20) = 1.57, p = 0.14 at 12 weeks], or the granular layer of the dentate gyrus [F(2 21) = 2.09, p = 0.15 at 6 weeks; F(2 20) = 2.93, p = 0.08 at 12 weeks]. In animals at 6 or 12 weeks of age, there were no significant birth group differences in terms of the number of pyramidal cells in the CA1 [F(2 21) = 0.18, p = 0.84 at 6 weeks; F(2 20) = 1.29, p = 0.30 at 12 weeks] or the CA3 [F(2 21) = 0.46, p = 0.64 at 6 weeks; F(2 20) = 1.62, p = 0.12 at 12 weeks] region. As regards the number of granule cells, no differences were observed among birth groups at 6 weeks of age [F(2 21) = 1.09, p = 0.36]; however, in animals at 12 weeks of age, there was a significant birth group difference [F(2 20) = 6.61, p = 0.01]. The *post-hoc* Bonferroni tests revealed that the number of granule cell was significantly reduced in the A group (by about 20%), compared with the corresponding values in the V and C groups (p<0.01 and p<0.05, respectively).

**Table 2:** Histological changes in the C-section with global asphyxia group.

Brain region	Age	Group	Number of principal cells	Volume (mm^3^)	Coefficient of error	Number of sections sampled
Dentate gyrus	6 weeks	V (n = 8)	766,541±41,273	1.449±0.054	0.064	8.4
		C (n = 8)	793,843±38,358	1.493±0.042	0.061	8.1
		A (n = 8)	717,811±30,219	1.328±0.076	0.065	8.8
	12 weeks	V (n = 8)	798,682±17,876	1.527±0.065	0.060	10.0
		C (n = 8)	766,541±32,293	1.406±0.048	0.062	10.4
		A (n = 7)	663,157±28,881* ^,^ †	1.351±0.038	0.069	10.4
CA1 region	6 weeks	V (n = 8)	288,922±10,195	0.742±0.030	0.070	14.3
		C (n = 8)	282,874±3,134	0.744±0.034	0.070	14.0
		A (n = 8)	290,282±11,940	0.718±0.029	0.071	13.9
	12 weeks	V (n = 8)	319,162±9,678	0.780±0.037	0.068	15.8
		C (n = 8)	320,371±20,752	0.740±0.032	0.066	15.6
		A (n = 7)	291,291±12,293	0.704±0.028	0.069	16.0
CA3 region	6 weeks	V (n = 8)	175,438±3,997	0.958±0.017	0.060	12.8
		C (n = 8)	177,057±3,534	0.957±0.020	0.059	13.0
		A (n = 8)	172,998±4,322	0.975±0.021	0.059	12.9
	12 weeks	V (n = 8)	192,234±4,142	0.977±0.022	0.058	13.6
		C (n = 8)	185,895±5,608	0.956±0.039	0.056	13.4
		A (n = 7)	186,969±4,327	0.926±0.085	0.059	13.6

The number of principal cells and the volume are given as means±SEM. Coefficient of error and the number of sections sampled are given as means. Note a significant difference: *p<0.01 vs. the V group; ^†^p<0.05 vs. the C group.

## Discussion

Twelve-week-old (adult) rats that had been exposed to intrauterine global hypoxia at birth after the C-section of anesthetized dams exhibited an increase in the amount of methamphetamine-induced locomotor activity, whereas similarly treated 6-week-old (adolescent) rats did not show such an increase in activity. This delayed occurrence of increased sensitivity to an indirect dopamine agonist may be comparable with the results of a previous study, which demonstrated an enhancement of amphetamine-induced locomotor activity at 56 days, but not 35 days in rats that had undergone global hypoxia at birth after the C-section of decapitated dams [22]. However, the increased sensitivity to a dopamine agonist observed in that study appears to have emerged earlier than in the present study (56 days [8 weeks] vs. 12 weeks), most likely because the more prominent hypoxic state was induced by decapitation in the previous study. Thus, the severity of hypoxia at birth may be a critical factor in the onset of dopaminergic sensitivity in the mature brain. Our results also demonstrated that transient global hypoxia at birth can induce an increase in extracellular dopamine levels in the 12-week-old nucleus accumbens after methamphetamine injection as measured by microdialysis and high-performance liquid chromatography. This finding is consistent with the general assumption that methamphetamine increases locomotor activity via enhancement of dopamine release in the nucleus accumbens [20].

Global hypoxia at birth did not affect the number of neurons in hippocampal areas CA1 and CA3 in 6- and 12-week-old animals. However, global hypoxia at birth was associated with a decrease in the number of dentate granule cells in the hippocampus at 12 weeks, but not at 6 weeks. The mature hippocampus is known to send outputs to the striatum, including to the nucleus accumbens [23], and it regulates behavioral changes induced by dopamine agonists, including amphetamine [11]–[14]. More specifically, we previously demonstrated that methamphetamine-induced locomotor activity is greatly enhanced by the bilateral and selective destruction of granule cells by colchicine in adult rats [24]. Since a lack of a change in the number of pyramidal cells in asphyxic animals is suggestive of the preservation of the function of these cells, the increased sensitivity to methamphetamine in the 12-week-old animals observed here may be associated with a reduced number of dentate granule cells in the hippocampus. Given the sequential nature of the hippocampus (i.e., information travels sequentially from the dentate gyrus to the CA3 to CA1 areas, and then to the subiculum in the hippocampus), a reduction in the number of granule cells may have resulted in modified or disrupted input from the dentate gyrus to the CA3 region, and may in turn have altered the subsequent efferent system from the hippocampus, which is mediated by the subiculum. The subiculum sends substantial glutamatergic output to the nucleus accumbens [23], [25], and there is evidence that glutamate can modulate dopamine release presynaptically [26], [27], although this relationship remains to be elucidated.

In this study, the animals delivered C-section without asphyxia responded to methamphetamine equal to animals born vaginally. The result seemed in contrast to the previous report by El-Khodor and Boksa [28], in which C-section with or without an added period of anoxia had increased amphetamine-induced behavioral response in 3-month-old adult rats. Although the reason for the difference between two studies is unknown, it might be due to differences in C-section procedures: while El-Khodor decapitated dams for the hysterectomy, we anesthetized dams by diethyl ether. The anesthetic used at C-section in this study might have prevented or reduced dopamine-mediated behavioral changes in adulthood induced by C-section. However, this is unlikely because Vaillancourt and Boksa [29] showed that adult (3-month-old) rats born by C-section from isoflurane-anesthetized dams, either with or without added anoxia, showed greater amphetamine-induced activity than controls born vaginally from un-anesthetized dams. Therefore, other differences in experimental designs between two studies may be underlying the inconsistent results. For instance, the psychostimulant used was different, i.e., d-amphetamine in El-Khodor's study vs. d-methamphetamine in ours. Systems used for the behavioral assessment were also different; El-Khodor used a chamber of 300×400×400 mm equipped with two photoelectric switches, while we used a chamber of 440×260×400 mm equipped with 144 pairs of photo sensors located at 5-mm intervals.

Although we had intended to measure the amount of extracellular dopamine in the nucleus accumbens in the microdialysis experiment, the probes appeared to be located partly in the caudate nucleus as well (Figure 2A). As reported in the study by Tani et al. [24], selective destruction of dentate granule cells by colchicine resulted in a marked increase in methamphetamine-induced Fos protein expression, a marker of neuronal activity, not only in the nucleus accumbens but also in other brain regions such as the dorsal striatum. Since the relative proportion of the probes located in the caudate nucleus did not differ by group status in this study, the finding of increased extracellular dopamine in asphyxic animals is reasonably considered to be due to the reduction of dentate granule cells.

In conclusion, the findings from the current study suggest that perinatal asphyxia may induce a reduction in the number of dentate granule cells in adulthood, which may account for the alteration in dopamine-associated behaviors. Such a mechanism may be a biological basis for the results of epidemiological studies [2]–[5] showing that obstetric complications are a risk factor for the development of schizophrenia. Exploring the molecular background behind the suppression of dentate granule cells may be helpful for gaining a better understanding of the late-onset emergence of manifestations of schizophrenia.
